# Effect of weekly or daily dosing regimen of Gefitinib in mouse models of lung cancer

**DOI:** 10.18632/oncotarget.19785

**Published:** 2017-08-02

**Authors:** Qi Zhang, Ruichao Li, Xu Chen, Sang Beom Lee, Jing Pan, Donghai Xiong, Jiaqi Hu, Mark Steven Miller, Eva Szabo, Ronald A. Lubet, Yian Wang, Ming You

**Affiliations:** ^1^ Cancer Center, Medical College of Wisconsin, Milwaukee, WI 53226, USA; ^2^ Department of Pharmacology and Toxicology, Medical College of Wisconsin, Milwaukee, WI 53226, USA; ^3^ Chemopreventive Agent Development Research Group, Division of Cancer Prevention, National Cancer Institute, Rockville, MD 20850, USA

**Keywords:** weekly, daily, intermittent, Gefitinib, lung cancer

## Abstract

Gefitinib showed response in phase II clinical trials and with better clinical response in lung cancer with activating mutations in the tyrosine kinase domain of the EGFR. Questions of toxicity and potential dosing regimens impede the use in a prevention setting. This study will provide scientific evidence for the utility of testing and comparing weekly and daily dosing regimens in clinical trials. We employed the adenocarcinoma (AD) and squamous cell carcinoma (SCC) models to compare the efficacy of Gefitinib in daily or weekly dosing regimens. We also assessed the effectiveness of Gefitinib in altering growth of the H3255 xenograft. Bioluminescent imaging (BLI) and tumor size was evaluated. Relative expression of phospho-EGFR, phospho-ERK and phospho-AKT in the xenograft were evaluated by Western Blot analysis. In the lung AD model, Gefitinib showed significant inhibition of tumor load when treated with weekly or weekly intermittent dosing regimens in AJ/p53*^val135/wt^*mice whereas a daily dosing regimen did not decrease the tumor load significantly. In the H3255-Luciferase xenograft model, weekly treatment demonstrated better inhibition than daily treatment. The weekly dosing regimen exhibited greater inhibition of phospho-EGFR, phospho-ERK and phospho-AKT than the daily dosing regimen, which may be correlated with the antitumor effects of the different dosing regimens. Weekly dosing with Gefitinib had similar or better efficacy than the daily dosing regimen in pre-clinical models of NSCLC. The data provide scientific evidences for the utility of testing and comparing weekly and intermittent dosing regimens in clinical trials.

## INTRODUCTION

Lung cancer is the leading cause of cancer-related deaths in the United States. In contrast to the steady increase in survival for most cancers, advances have been slow for lung cancers, for which the 5-year relative survival rate is currently 18% [1]. About 85% of lung cancer is histologically classified as non-small cell lung carcinoma (NSCLC) [2]. NSCLC presents many clinical challenges, and chemoprevention is a potentially important approach to reduce this deadly disease [3]. In recent years, our understanding of the driving events at the molecular level in NSCLC formation and maintenance has improved dramatically. This knowledge opens new opportunities in gene targeting approaches against activated kinases [4]. Lung cancer development is associated with a number of molecular abnormalities. Targeting specific molecular mutations that are unique to lung tumor cells is a potential development in the treatment of lung cancer with possible applicability to prevention strategies as well.

One of the most promising targets identified in general is epidermal growth factor receptor (EGFR). The EGFR pathway was shown to be associated with a variety of important cellular pathways [5]. The EGFR pathway is activated by either mutation or overexpression in a wide variety of cancers. Both small-molecule EGFR inhibitors and anti-EGFR antibodies have been developed and approved for clinical use in the treatment of advanced NSCLC as monotherapy following failure of chemotherapy [6]. Most of the initial clinical trials with the EGFR inhibitors have used individuals with metastatic disease who have failed standard chemotherapy as monotherapies. However, a limited number of studies in earlier stages of cancer have shown more striking efficacy, implying that this class of compounds might be more useful in earlier clinical stages and for cancer prevention [7].

Gefitinib (Iressa, ZD1839), a competitive EGFR tyrosine kinase inhibitor, was selected from a variety of substituted 4-(3-chloroanilino)-quinazolines as the most effective in inhibiting tyrosine kinases activity [8]. Many *in vitro* and *in vivo* studies have shown that Gefitinib can inhibit the growth of cancer cells and suppress the growth of tumors [9, 10]. Gefitinib has been approved for clinical use in the treatment of advanced NSCLC as monotherapy following failure of chemotherapy [6]. Gefitinib was also shown response in phase II clinical trials [11] with better clinical response in lung cancer patients harboring activating mutations in the tyrosine kinase domain of the EGFR [12].

The toxicity of many of the newly developed targeted agents, including EGFR inhibitors, is a major concern for use in the adjuvant setting and particularly in primary cancer prevention [13]. A recent study on an EGFR inhibitor, erlotinib, has shown that daily and weekly dosing were equally effective for preventive/therapeutic efficacy in a rat mammary cancer model [14]. In addition, clinical data with both Erlotinib and Gefitinib showed that weekly dosing results in decreased toxicity [15, 16]. Therefore, employing weekly dosing of the EGFR inhibitor may yield strong efficacy with lower side effect. One additional question might be whether these altered dosing schedules could still retain efficacy in an intermittent schedule of 9 weekly doses.

In the present study, we investigated the effects of weekly and daily dosing on AD and SCC mouse modes and the mechanism of inhibition. Treatment with weekly Gefitinib resulted in equal or better inhibitory effects compared with daily dosing. Using the H3255-Luciferase xenograft model, we also compared the bioluminescence and tumor size of different treatment schedules. The results indicated that weekly dosing exhibited a more pronounced inhibition than a daily dose regimen. Tumor volume was also measured and the weekly dosing schedule demonstrated a more marked inhibition compared with daily treatment. This was associated with decreased expression of phosphorylated EGFR, extracellular regulated kinase (ERK) and v-akt murine thymoma viral oncogene homologue (AKT) signaling molecules. These data suggest that a weekly dose schedule is effective in the prevention and treatment of carcinogen-induced lung cancers. Our study provides a scientific rationale for testing such regimens in human cancer prevention trials.

## RESULTS

### Inhibitory effect of Gefitinib on lung tumor multiplicity and load in B(a)P-induced A/J mice

In this study, we investigated the inhibitory effect of Gefitinib by daily, weekly and intermittent dosing on B(a)P-induced AD formation. To monitor the well-being of the animals, the body weight was measured weekly. Body weights were in the normal range when compared to their corresponding vehicle-control mice (Supplementary Figure 1). In addition to determination of BW, the well-being of each mouse was also monitored twice a week by evaluating their general appearance (skin, hair, eyes, nose and breathing); clinical signs such as diarrhea and bleeding; and behavioral changes affecting eating or drinking. There were no obvious abnormalities in these parameters during the course of the study when compared to their corresponding vehicle-control mice. We also measured the Alanine Aminotransferase (ALT) and Aspartate Aminotransferase (AST) level in blood and did not observe any differences between these groups (data not shown).

**Figure 1:**
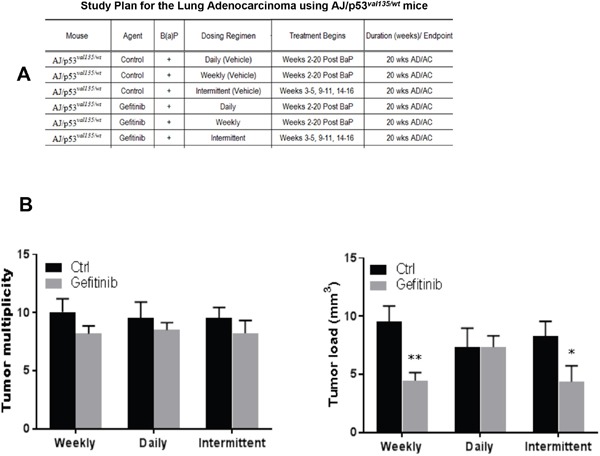
Effects of Gefitinib treatment in B(a)P-induced lung tumorigenesis in AJ/p53^val135/wt^ mice The results show that the multiplicity and load of tumors in mice treated with Gefitinib decreased compared with carcinogen treated control mice. **(A)** Study plan for the lung adenocarcinoma using AJ/p53*^val135/wt^* mice. **(B)** Lung tumor multiplicity, lung tumor load, *p<0.05, **p<0.01, compared with control group (n=18).

The lung tumor incidence was 100% in all groups of mice. Lung tumor development was estimated quantitatively by tumor multiplicity and tumor load. As shown in the Figure 1, statistically, Gefitinib did not exhibited significant effect on lung tumor multiplicity with all three treatment protocols. However, weekly or intermittent dosing regimens showed a significant inhibition of tumor load. Animals treated with weekly intermittent dosing of Gefitinib significantly decreased tumor load by 53.0% (P<0.01) or 47.2% (P<0.05), respectively, which demonstrating the weekly dosing schedule had better inhibitory effects than daily dosing in B(a)P-induced AD model.

### Inhibitory effect of Gefitinib in NTCU-induced lung SCC mouse model

**Figure 2:**
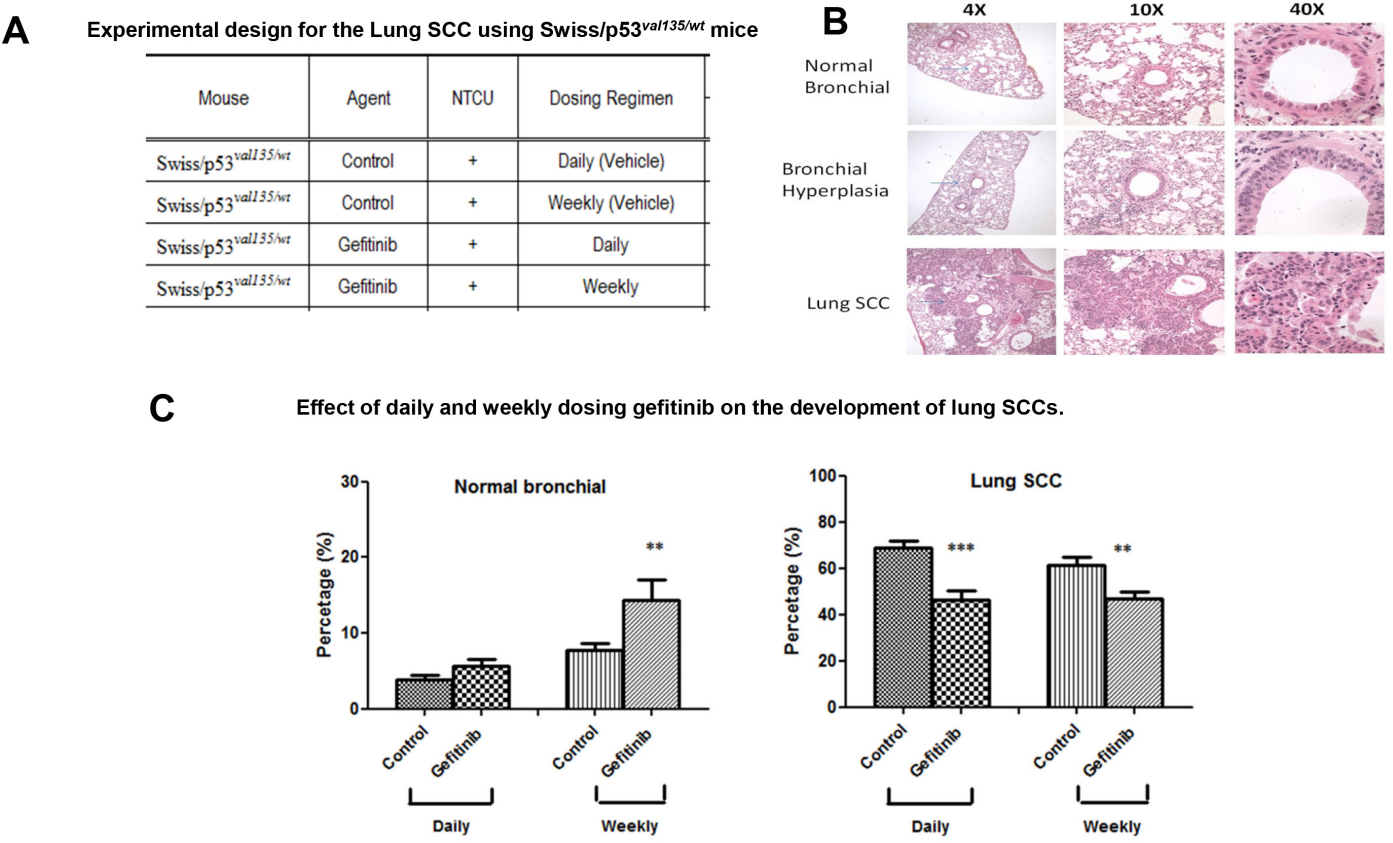
Effect of daily or weekly treated Gefitinib on lung SCC development **(A)** Experimental design for the lung SCC using Swiss/p53*^val135/wt^* mice. **(B)** Typical H&E staining for NTCU-induced squamous cell lung carcinoma (lung SCC), bronchial hyperplasia, as well as normal bronchial epithelium are shown at 4x, 10x, and 40x. Arrow indicates the lesions. **(C)** Effect of daily or weekly treated Gefitinib on lung SCC development based on the percentage of normal and SCC histopathology (n=20) **p<0.01, ***p<0.001.

We counted the lesions in the following categories: invasive SCC, carcinoma *in situ* (CIS), and bronchial hyperplasia/metaplasia as well as normal epithelium from the H& E-stained sections of each lung. All cross-sectional cuts of bronchioles were counted on all slides. Typical histopathological lesions are shown in Figure 2. In daily control NTCU-treated animals, the distributions of lesions are as follows: normal bronchi (3.8±0.6%), bronchial hyperplasia (27.2±2.6%), and SCC *in situ* (69.0±2.9%). Animals treated with daily or weekly dosing of Gefitinib significantly decreased SCC by 46.4% (p<0.01) or 46.9% (p<0.01), respectively. The percentage of normal lung tissue increased with Gefitinib treatment, which indicates that both daily and weekly dosing can inhibit the progression of normal lung epithelium to lung SCC.

### Biomarker analyses in H3255-Luciferase xenograft model

**Figure 3:**
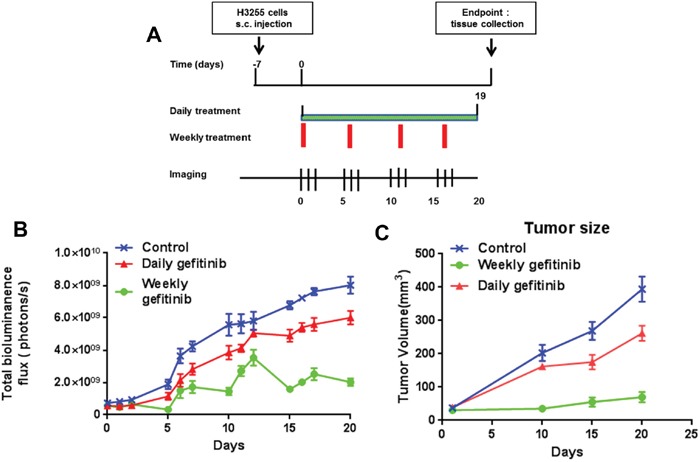
Effect of daily and weekly Gefitinib in H3255-Luciferase mice Mice were injected s.c. in each front flank with 100 μl of cell suspension containing five million H3255-Luciferase cells and Matrigel (BD Biosciences, Beit Haemek, Israel, 20% v/v) in PBS. One week after injection, mice were divided into 3 groups with 6 mice per group. Tumors were imaged 4 min post 200 μL (15 mg/mL) intraperitoneal injection of D-luciferin (Xenogen Corporation). Isoflurane anesthetized mice were imaged at indicated time point using the Lumina IVIS-100 *in vivo* Imaging System (Xenogen Corporation). Regions of interest were created and measured as area flux, defined by radiance (photons per seconds per square centimeter per steradian) according to the manufacturer's calibration (Xenogen Corporation). **(A)** Experimental design. **(B)** BLI of daily and once every 5-day treatment in mice. **(C)** Tumor measurement of daily and weekly treatment.

We examined the benefit of different dosing regimens using the H3255 xenograft model. Mice were repeatedly administered daily or once every 5-day doses of Gefitinib over a period of 20 days. The daily dose of Gefitinib in mice (40 mg/kg body weight /day) was roughly equivalent to the human equivalent dose of 211 mg based on FDA scaling factors (Supplementary Table 1). The effectiveness of Gefitinib in preventing the development of the H3255 xenograft was assessed by orally administering drug once daily 5 days per week or once every 5-day at a dose of 200 mg/kg body weight. The effect of the different dosing schedules was measured by bioluminescence (BLI). As shown in Figure 3, significant decreases in tumor growth were observed at the earliest time point measured after initiation of Gefitinib on day 5 as determined by BLI. The once every 5-day dosing protocol exhibited a greater effect on tumor growth than daily treatment. Increasing BLI was observed in the once every 5-day group two days after treatment. However, the increase was much lower than that observed in the daily dosing group. Interestingly, the BLI of the once every 5-day group showed periodic oscillations. At the same time, tumor volume was also measured (Supplementary Figure 2); once every 5-day dosing resulted in greater inhibition compared with daily treatment. These experiments collectively demonstrate that treatment with higher doses of Gefitinib with less frequent drug administration were more effective at treating H3255-Luciferase xenograft.

### The relative expression of total phospho-EGFR, phospho-ERK and phospho-AKT Levels in tumor samples

**Figure 4:**
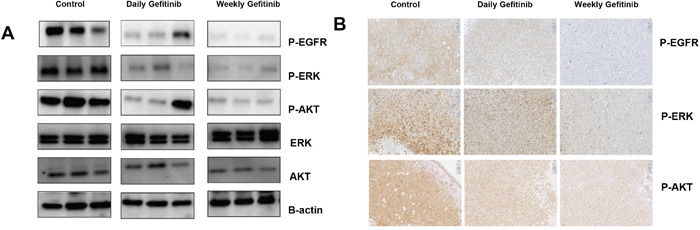
Weekly dosing with Gefitinib exhibits greater inhibition of phospho-EGFR, phospho-ERK and phospho-AKT compared with daily dosing **(A)** Western blot analyses of phospho-EGFR, phospho-ERK and phospho-AKT in tumor samples. Mice were treated with Gefitinib with the daily and weekly dosing protocols. β-actin was used as the internal control. **(B)** Immunohistochemical staining of phospho-EGFR, phospho-ERK and phospho-AKT in xenograft tumor sections from each group indicating inhibition with daily and weekly treatments.

To confirm that Gefitinib was affecting its intended molecular targets in these mice, the relative expression of total phospho-EGFR, phospho-ERK and phospho-AKT levels in the four investigated NSCLC tumors was evaluated by Western blot analysis. As shown in Figure 4A, the expression of phospho-EGFR, phospho-ERK and phospho-AKT was somewhat decreased in mice treated with daily Gefitinib treatment, but were dramatically reduced in animals treated with the weekly dosing protocol. In contrast, ERK and AKT were not significantly different from the normal group. We next confirmed the Western blot results using immunohistochemical staining in xenograft tumor tissues. We found that the levels of phosphorylated EGFR, AKT and ERK were decreased compared to the control and daily dose groups (Figure 4B). The results demonstrated that weekly dosing had greater inhibitory effects on the levels of phospho-EGFR, phospho-ERK and phospho-AKT, which may account for the enhanced antitumor activity of the weekly dosing relative to the daily dosing protocol.

### Daily Gefitinib treatment showed enhanced contact hypersensitivity response compared with weekly treatment

**Figure 5:**
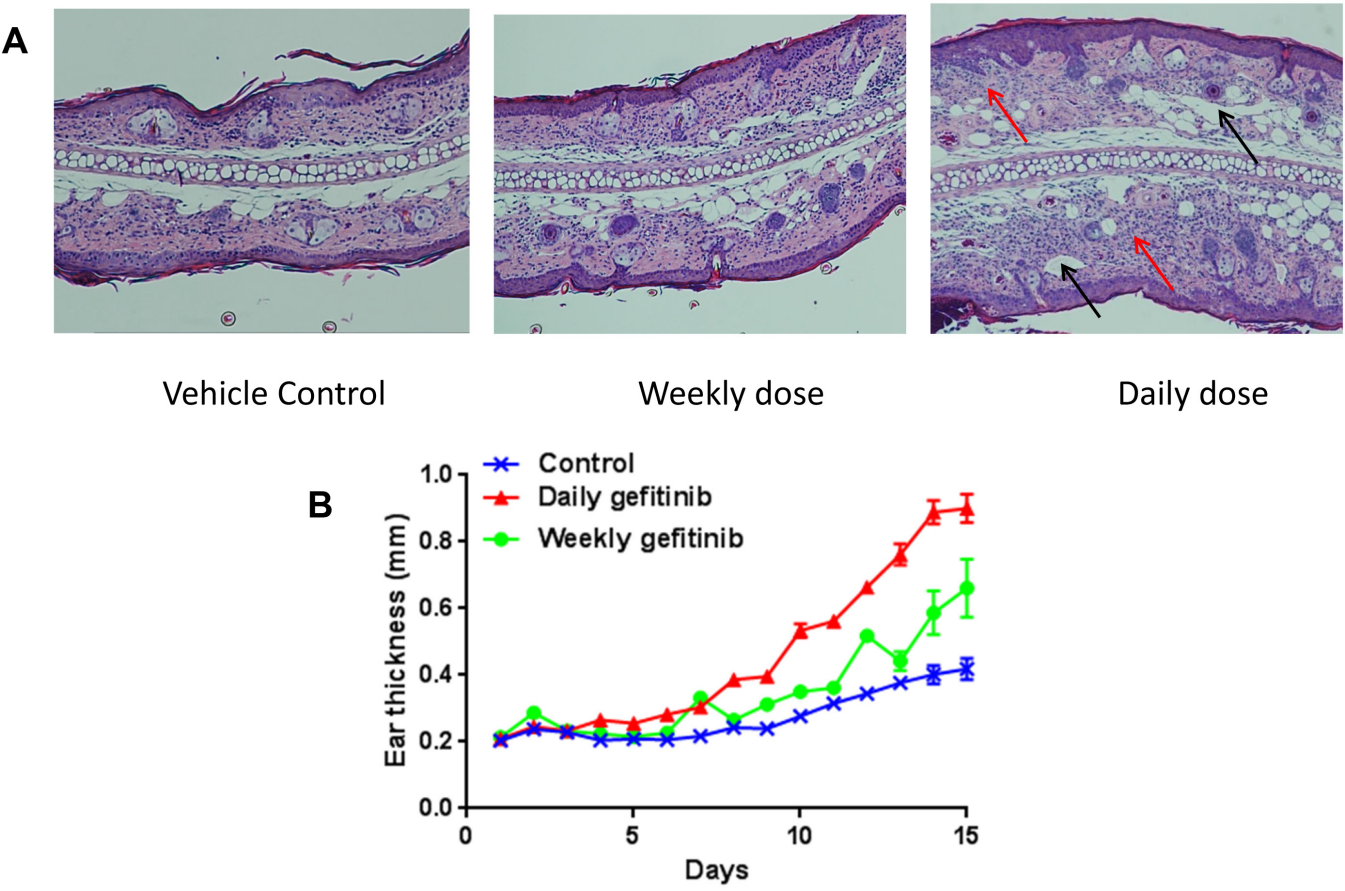
Daily treatment with Gefitinib increases DNFB-induced contact dermatitis Eighteen A/J mice were sensitized by application of 15 μl of 0.2% 2,4-domodtrpfluorobenzene (DNFB) in acetone/olive oil (4/1) on the upper side of the right ear of each animal. Two days later, sensitized mice were randomly divided to control, daily treatment and once every 5-day treatment groups. Ear thickness was measured 1.5 hour after challenge for 15 days. All mice were sacrificed after 15 days, and the ears were harvested for hematoxylin & eosin staining. **(A)** Representative H&E histology 15 days after DNFB irritation correlates with the effects of daily and weekly treatment. In control and once every 5-day treatment groups, sensitized mice had similar mild dermal inflammatory cell infiltration. In the daily treatment group, the ear was markedly thickened with edema and inflammatory infiltration in the dermis. Heavy infiltration of polymorph nuclear neutrophils (red arrows) and dilated lymphatics (black arrows) can be found. **(B)** Ear swelling responses for daily and once every 5-day treatment.

To investigate the difference of daily and once every 5-day treatment of Gefitinib, we tested the contact hypersensitivity response in mice (Figure 5). Mice treated with Gefitinib daily showed a marked increase in contact hypersensitivity response to DNFB compared to the once every 5-day treatment group, which was associated with a more extensive inflammatory infiltrate in the ears of mice treated daily. These results showed mice treated less frequently with a larger dose of Gefitinib exhibited less toxicity compared with mice that received daily treatment.

## DISCUSSION

EGFR has been a major target of interest in cancer treatment for years due, in part, to it's over expression in a wide variety of human cancers (e.g. lung, head and neck, breast, colon, pancreas) [21]. Inhibitors of EGFR, both small molecule inhibitors and antibodies, have progressed into the clinic. The area where EGFR inhibitors have the clearest clinical applicability is in the subset of lung AD which has mutations in the EGFR, in which it is strikingly effective. However, there are a variety of potential cancers in which EGFR inhibitors are less effective but clinically significant in which its toxicities (primarily acneiform rash) are a significant drawback. These include 1) Pancreatic cancer in combination with standard chemotherapy; 2) Familial adenomatous polyposis (FAP) in combination with sulindac where it significantly inhibits polyp formation. 3) NSCLC, without EGFR mutations, in which it has been shown to inhibit tumor progression; 4) Head and neck cancers combined with radiation.

Gefitinib, an EGFR tyrosine kinase inhibitor, is an effective single-agent therapy in previously treated NSCLC [22]. However, some studies showed Gefitinib has no *benefit* to *chemotherapy*, possibly due to incomplete inhibition of the EGFR tyrosine kinase with daily Gefitinib therapy and/or the development of resistance to chemotherapy [23, 24]. Intermittent high-dose EGFR has shown the potential to overcome these theoretical limitations of daily dosing in several studies [14, 25, 26].

In this study, we set up three different dosing regiments in two different prevention protocols to compare the chemopreventive efficacy of daily or weekly Gefitinib, as well as its side effects. The first protocol uses B(a)P-induced lung AD in A/J mice that mimic the histopathology and stages of tumor progression with human lung AD. The second model utilized NTCU-induced lung SCC in Swiss/p53*^val135/wt^*mice, which is a promising model for mechanistic studies examining SCC development for preclinical screening of potential therapeutic or preventive agents and for the study of genetic susceptibility to SCC induction [18]. Our results demonstrated that weekly treatment with Gefitinib was more effective than daily treatment in the lung AD model. Intermittent dosing was less effective than weekly dosing when comparing tumor load. To our knowledge, this is the first time dosing regimens were compared in multiple mouse lung cancer models. This finding agrees with our prior studies showing that Gefitinib is effective in these models [[10] and unpublished data]. In the squamous cell model, although Gefitinib demonstrated statistically significant efficacy relative to untreated mice, there were minimal differences between daily and weekly dosing. We might have seen more striking efficacy in these experiments if we had chosen to employ a higher dose of Gefitinib but in fact we had determined to use a dose that was equivalent to the human dose based on normal FDA scaling factors.

Side effects are a critical aspect of treating patients with anticancer drug [27]. The most common side effect of Gefitinib is skin reactions [28], which might limit their acceptability in a prevention setting. In our study, we monitored well-being of each mouse on regular bases as described in the Material and Method, as well as blood levels of ALT and AST. We did not find obvious abnormalities in these parameters during the course of the study. In an attempt to attenuate cumulative toxicity, we set up an intermittent treatment group in the lung AD models. The intermittent schedules were less effective as weekly treatment in decreasing tumor load, but had better efficacy compared with daily treatment. EGFR inhibitors were reported to enhance immediate contact dermatitis and skin hypersensitivity in the 2,4-dinitro uorobenzene (DNFB) induced mouse skin model [28–31]. In our study, daily treatment with Gefitinib showed enhanced contact hypersensitivity response compared with weekly treatment and DNFB-induced control group. This experiment indicated that weekly treatment may have less toxicity, as measured by skin reaction, compared with daily treatment.

The most striking result we obtained was in the human xenograft containing a mutation in EGFR^L858R^, which is the most common single mutation in EGFR mutant lung cancers [32]. As expected, daily dosing with Gefitinib was moderately effective in reducing tumor growth. Interestingly, the weekly dosing protocol was profoundly effective. This result raises an interesting question regarding treatment. Daily dosing with Gefitinib can cause the initial regression of tumors in most patients with primary EGFR mutant lung cancer. However, although most individuals have tolerable inhibitor related toxicity certain individuals suffer from dose-related toxicities severe enough to require either that lower doses of agent is utilized or a drug holiday be implemented. Why the weekly dosing is more effective than the daily dosing? One possibility is weekly dosing had greater inhibitory effects on the levels of phospho-EGFR, phospho-ERK and phospho-AKT, which may account for the enhanced antitumor activity. Another possibility might be due to the reduced drug resistance related to the less frequent administration of the drug. The present studies would appear to support the use of weekly dosing to reduce toxicity in sensitive patients. It might also warrant its use in individuals with Asian or Latino ancestry who has identified lesions based on Computed Tomography (CT) scans, since these two populations have a high incidence of EGFR mutations [33, 34].

In summary, the present study suggests that weekly dosing with Gefitinib may be more effective than daily dosing while also reducing the associated side effect. This finding opens the possibility of using EGFR inhibitors where it has shown efficacy but where toxicity may be significantly limiting e.g. pancreas, polyps in FAP. Also it clearly opens up its use in Asian or Latino population with lesions based on scanning and raises the potential for the use of EGFR inhibitors in a prevention setting in these populations.

## MATERIALS AND METHODS

### Reagents and animals

Benzo(a)Pyrene (B(a)P, 99% pure) and tricaprylin were purchased from Sigma Chemical Co. (St. Louis, MO). B(a)P was prepared immediately before use in animal bioassays. *N*-nitroso-tris-chloroethylurea (NTCU) was purchased from Toronto Research Chemicals, Inc. (Toronto, Canada). Gefitinib was provided by the NCI Chemical Repository. P53 mutant A/J mice or p53 mutant NIH Swiss mice, which carry the A135V mutation, were used for our study. The A/J mice (wild type) were purchased from the Jackson Laboratory (Bar Harbor, Maine). The NIH Swiss mice (wild type; short as Swiss mice) were purchased from Charles River (Frederick, Maryland). The p53 mutant mice (p53*^val135/wt^*) were crossed to A/J mice (wild type) or Swiss for over 10 generations to move the mutant alleles to different genetic backgrounds and are referred to as AJ/p53*^val135/wt^*or Swiss/p53*^val135/wt^* in the current study. Mice were treated by oral gavage with an 18-gauge gavage needle with a final volume of 0.2 ml per mouse. Control animals were treated with an equal volume (0.2 ml) of corn oil (vehicle) throughout the study. Female athymic nude mice (Crl:NU(NCr)-Foxn1nu) were purchased from Charles River.

### Chemopreventive study of daily and weekly treatment of Gefitinib

Two animal studies were carried out. In both study, all mice were monitored for their well-being, including body weight (BW), general appearance (e.g., skin, hair [rough hair coat], eyes, nose, breathing, locomotion), clinical signs (e.g., rapid weight loss, diarrhea, bleeding), behavior changes (e.g., eating, drinking, or lethargy), breathing abnormalities, and posture (e.g., hunched posture or body hunching).

In experiment 1, mice were given a single i.p. injection of B(a)P at 100 mg/kg body weight in 0.2 ml of tricaprylin. Six-week-old AJ/p53*^val135/wt^* mice were randomized into 6 groups with 18 mice per group (Figure 1A). Gefitinib was freshly prepared in Mazola corn oil before gavage administration. Oral gavage was administered once a day 5 times a week for the duration of the study. Control animals were treated with 0.2 ml corn oil throughout the study. The treatment was begun 2 weeks after the B(a)P injection and continued for 18 consecutive weeks. The daily dose of Gefitinib was 80 mg/kg body weight; oral gavage once a day, 5 days a week. The weekly dose of Gefitinib was 400 mg/kg body weight; oral gavage once weekly. The intermittent dosing of Gefitinib was 400 mg/kg body weight; oral gavage once weekly on weeks 3, 4, 5, 9, 10, 11, 15, 16, 17 with final sacrifice at week 20.

In experiment 2, Swiss/p53*^val135/wt^* mice at 8 weeks of age were given the first dose of NTCU and this time point is noted as “week 0”. The dorsal skin of each mouse was shaved 24 to 48 hours prior to the first dose of NTCU. For the application of NTCU, 100-microliter drops of 0.04 M NTCU was applied to the shaved skin with a micro-pipette. This process was repeated twice a week with a 3.5-day interval for 30 consecutive weeks (Figure 2A).

Two weeks after the first dose of NTCU when mice were ∼10 weeks old, mice were divided into the 4 groups and treated with Gefitinib using both daily and weekly dosing regimens. Gefitinib daily (80 mg/kg body weight/day i.g., 5 days a week) and weekly dosing (5 × 80 mg = 400 mg/kg body weight i.g. oral gavage once weekly) were used.

Animals were housed with wood chip bedding in an environmentally controlled, clean-air room with a 12-hour light-dark cycle and a relative humidity of 50%. Drinking water and diet were supplied *ad libitum*. The study was approved by the Institutional Animal Care and Use Committee at the Medical College of Wisconsin.

For both experiments, body weight was recorded weekly. Mice were euthanized by carbon dioxide (CO_2_) asphyxiation. Lungs of each mouse were fixed in zinc formalin solution overnight then stored in 70% ethanol [17]. For the lung AD model, the fixed lungs were evaluated under a dissecting microscope to obtain the surface tumor count and individual tumor diameter. Tumor volume was calculated based on the formula: V = 4πr^3^/3. The total tumor volume in each mouse was calculated from the sum of all tumors. Tumor load was determined by averaging the total tumor volume of each mouse in each group. For the SCC model, mice were euthanized by CO_2_ asphyxiation at 32 weeks after the first dose of NTCU treatment. Lungs from all mice were fixed in Zinc formalin overnight then stored in 70% ethanol. Histopathological evaluation of the lung tumors was carried out using the following method [18]: approximately 100 serial tissue sections (4 μm each) were cut from formalin fixed lung, and one in every 20 sections (approximately 100 μm apart) was stained with hematoxylin and eosin (H&E). The lung SCCs area/lung lobe area ratio was evaluated using NanoZoomer Digital Pathology Virtual Slide Viewer software (Hamamatsu Photonic Co.). H&E-stained slides were scanned with the NanoZoomer HT slide scanner (Hamamatsu Photonics), and virtual slides were analyzed and quantified.

### Cell establishment

H3255 is a human NSCLC cell line which contains an EGFR^L858R^ mutation. To establish H3255-Luciferase expressing cells, LV-CMV-Puromycin-firefly luciferase was transduced into H3255 cells according to the manufacturer's protocol. Briefly, 1 × 10^5^ cells were plated in 6-well plates and, 24 hours later, the media was replaced with transduction media containing lentivirus expressing puromycin luciferase fusion protein and polybrene (8μg/ml). Forty-eight hours after transduction, the infected cells were selected with puromycin (2 μg/ml) for 3 days and pooled cells stably expressing luciferase were used in the study.

### Xenograft mouse experiment

Six week old female athymic nude mice (Crl:NU(NCr)-Foxn1nu, Charles River) were used for this study. After an esthesia using isoflurane, mice were injected s.c. in each front flank with 100 μl of cell suspension containing five million H3255-Luciferase cells and Matrigel (BD Biosciences, Beit Haemek, Israel, 20% v/v in PBS. One week after injection, mice were divided into 3 groups with 6 mice per group. Control animals were treated with 0.2 ml corn oil throughout the study. Daily (40 mg/kg body weight/day i.g.) and weekly (5 × 40 mg = 200 mg/kg body weight i.g. oral gavage 1 time per 5 days) Gefitinib doses were used. Tumor volume was calculated by the formula 0.52×length×width^2^.

### Bioluminescence imaging (BLI)

Briefly, tumors were imaged 10 min post 200 μL (15 mg/mL) intraperitoneal injection of D-luciferin (Xenogen Corporation). Isoflurane anesthetized mice were imaged at indicated time points using the Lumina IVIS-100 *in vivo* Imaging System (Xenogen Corporation). Regions of interest were created and measured as area flux, defined by radiance (photons per second per square centimeter per steradian) according to the manufacturer's calibration (Xenogen Corporation) [19].

### Western blotting

Xenograft tumor specimens were lysed in RIPA buffer (Thermo Scientific) containing phosphatase-proteinase inhibitors (Thermo Scientific) at 4°C and immediately homogenized with Tissue Lyzer (Qiagen) for 5 min. Lysates were then incubated for 10 min on ice and centrifuged for 5 min at 13,000 X *rpm*. The supernatant was first mixed with BCA reagent (Thermo Scientific) to measure protein concentration, and then equal amounts of protein lysates were mixed with Laemmli buffer and boiled for 5 min at 95°C. Twenty micrograms of proteins from each sample were electrophoresed in 4∼15% SDS-PAGE. Size standards from 10 to 200 kDa (BioRad) were included in each gel. After transfer to a PVDF membrane at 80V for 2 hours, membranes were blotted with 5% non-fat dry milk in TBST for 30 min and incubated with primary antibodies (Cell Signal) in TBST overnight at 4°C. Membranes were then washed three times for total of 15 min in TBS-T to remove unbound antibody, and then incubated for 1 hour with secondary antibodies (Santa Cruz Biotechnology). The following antibodies were used: rabbit phospho-EGFR(#3777s), EGFR(#4267s), phospho-AKT (#4060s), AKT (#9272s), phospho-ERK1/2 (#4370s), ERK1/2 (#9012s) (Cell Signaling Technology, Danvers, MA), and mouse anti-Actin (sc-81178) (Santa Cruz Biotechnology, Santa Cruz, CA).

### Histopathology analysis

Immunohistochemistry (IHC) analysis was performed to analyze tumor histology. All slides were deparaffinized in xylene and rehydrated in gradients of ethanol. Microwave antigen retrieval was carried out for 20 minutes in citrate buffer, pH 5.0–6.0. Primary antibody was diluted in DaVinci Green (BioCare) and incubated at 4°C overnight. Secondary antibody was diluted in phosphate buffered saline tween-20 (PBST) and SA-HRP (1:800) was then applied to the sections.

### Toxicity assessment

To monitor the well-being of animals, the body weight (BW) was measured weekly. In addition to determination of BW, the well-being of each mouse was also monitored twice a week by evaluating their [i] general appearance (skin, hair, eyes, nose, breathing, locomotion); [ii] clinical signs, such as diarrhea, bleeding; and [iii] behavioral changes affecting eating or drinking.

### Contact hypersensitivity assay

A/J mice (Jackson lab) were sensitized by application of 15 μl of 0.2% 2,4-domodtrpfluorobenzene (DNFB) (Sigma-Aldrich, St louis) in acetone/olive oil (4/1 v/v) on the upper side of the right ear in each animal. Two days later, sensitized mice were randomly divided to 3 groups, 1) control group; 2) daily Gefitinib treatment 5 mg/ml; and 3) weekly Gefitinib treatment 25 mg/ml. In selected groups, 10 μl of Gefitinib dissolved in DMSO/absolute ethanol (1/1 v/v) was applied on each side of right ear every 5 day in the weekly treatment group or every day in the daily treatment group. Control group was treated with vehicle. Thirty minutes after treatment, 0.2% DNFB was applied to the ear. Ear thickness was measured 1.5 hours after each challenge with DNFB, mice were sacrificed after 15 challenges, and the ears were removed and fixed for hematoxylin & eosin staining [20].

### Statistical analysis

ANOVA (analysis of variance) was used to test whether there was any significant difference across the different comparing groups. Once rejected the null hypothesis that there was no difference in the group mean, the “post-hoc” Tukey's test was used to identify what treatment differ. Adjusted p values from Tukey's test were given for pairwise comparisons to account for the multiple comparisons problem. * P < 0.05, ** P < 0.01, and *** P < 0.001 were considered statistically significant.

## SUPPLEMENTARY MATERIALS FIGURES AND TABLE


