# Supplementary material for: Effect of weekly or daily dosing regimen of Gefitinib in mouse models of lung cancer
Source: Oncotarget. 2017 Aug 2;8(42):72447–56. doi: 10.18632/oncotarget.19785 (PMC5641144; doi:10.18632/oncotarget.19785)
Supplement: Supplementary file 1 [file oncotarget-08-72447-s001.pdf]

Effect of weekly or daily dosing regimen of Gefitinib in mouse models of lung cancer

SUPPLEMENTARY MATERIALS

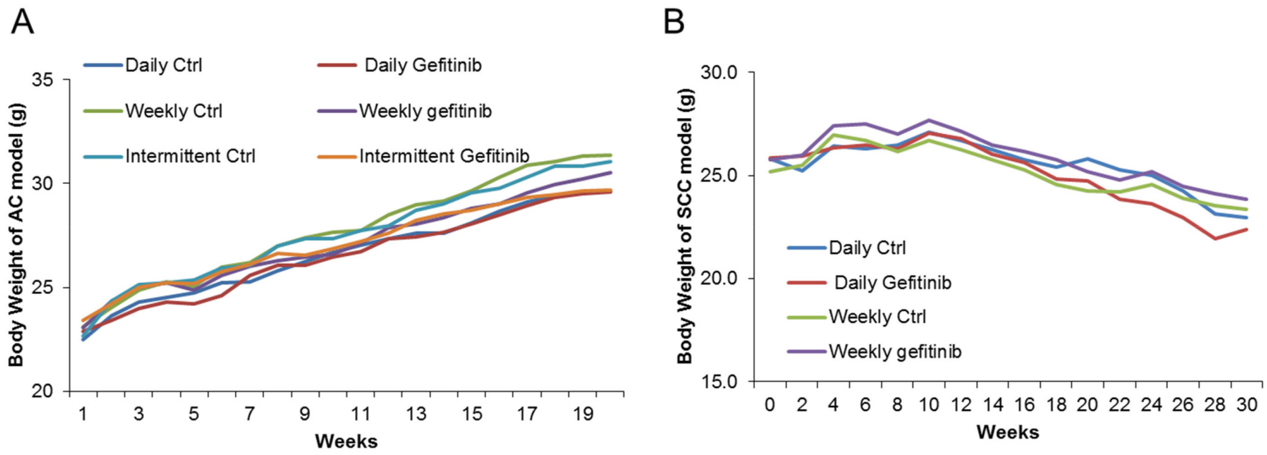

Supplementary Figure 1: Body weight of AC model and SCC model.

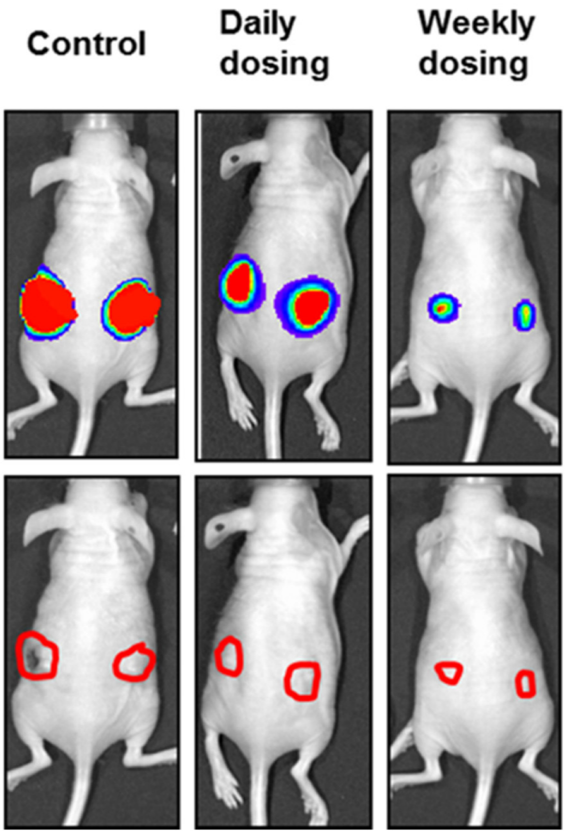

Supplementary Figure 2: Representative pictures of BLI and photograph for H3255 nude mice.

Supplementary Table 1: Conversation of mouse dose in this study to human equivalent dose

Gefitinib dose in mouse (mg/kg)	Human equivalent dose (mg)
40	211
80	422
200	1058
400	2110
